# Notes and News

**Published:** 1889-01-19

**Authors:** 


### Notes and News.

Collected and Communicated.

The patients' entertainment at St. Mary's Hospital on January 10th was a great success, Mr. Charles Collette being encored again and again. About one hundred and twenty patients were present, as well as a large number of nurses and others in the employ of the hospital.

The Christmas party at the East Sussex Hospital does not seem to have been a perfect success. The following announcement appears in a local paper: "The matron desires us to state that she would be pleased if those who took the children's toys away by mistake would return them on Wednesday evening next."

The General East-end Tradesmen's Association has held its annual meeting in aid of the London Hospital. During the last year they have subscribed £494 to this hospital. It is very pleasant to see the interest the inhabitants of the East-end take in the London Hospital: they thoroughly appreciate the good work it does, and many of them show their appreciation in a solid form.

At the children's treat at the Jenny Lind Infirmary, Norwich, the pleasant little hospital was quite a bower of greenery. It was touching to see the initials of the late Lady-Superintendent side by side with those of Miss Wainwright, who now occupies that position. It showed that Miss Peter is not forgotten in her former sphere of work. The little patients had a merry evening, and all received pretty gifts from Father and Mother Christmas.

Amongst the chief new hospitals opened in 1888 are the following: Birmingham and Midland Skin and Lock, Birmingham Workhouse Infirmary, Cromer Cottage Hospital, Coldstream Cottage Hospital, Faversham Cottage Hospital, Faversham Infectious Hospital, Gateshead Chil-Children's Hospital, Great Northern Central Hospital, Holloway, N., Victoria Cottage Hospital (Essex), Lynn Cottage Hospital: Liverpool City Infectious Hospital, Southend Victoria Hospital, Swindon Victoria Hospital, and Walker Infectious Hospital—thirteen in all.

The annual meeting of the subscribers to the Edinburgh Provident Dispensary was held on December 27th, Dr. Alison presiding. During the past year the total number of cases was 8,216, which necessitated at least 18,000 consultations and 12,000 visits. Since the opening of the institution nearly 60,000 patients have received advice and medicine. This includes nearly 3,000 midwifery cases, and in this large number only four deaths have occurred. The abstract of accounts showed a balance in the treasurer's hands of £11 7s. Subscriptions and donations amounted to £106, as against £89 last year. The chairman, in moving the adoption of the report, spoke of the usefulness of the Samaritan Society, the necessity that existed for thoroughly trained and qualified nurses, the benefits conferred by the dispensary, and the great indebtedness of the community to Dr. Urquhart, the medical men, and the nurses, all of whom gave their services ungrudgingly and freely.

The chief enlargements in 1888 of hospitals and dispensaries have occurred at Bristol Dispensary, Bradford Fever Hospital, Carnarvonshire Infirmary, Hereford General Infirmary, Lewes Infirmary, Manchester Clinical Hospital, Portsmouth and Gosport Hospital, Sunderland Infirmary, and Tottenham Cottage Hospital. Certainly the old year was one of great activity in the hospital world, and the new year will have its work cut out to efficiently provide for the new needs thus created.

The illustrated papers have been turning their attention to hospital matters very largely lately. Last week's *Lady's Pictorial* had sketches drawn at the Royal Free Hospital, the *Graphic* of January 5th had a page devoted to the Italian Hospital, and the *Lady's Pictorial* of that date had sketches of the children's fête at the Evelina. The *Graphic* of December 15th had an illustration of the nurses' conversazione at the Grosvenor Gallery, and also a good likeness of Lady Dufferin.

The annual report of the Devonshire Hospital, Buxton, has just been issued, and tells a tale of continued good work. The majority of the patients suffer from rheumatism, and that often of a chronic character; so that to have discharged 2,272 patients as much improved, and only 58 as no better, and six deaths, is a splendid report. The committee acknowledge a welcome donation of £100 from the Viscountess Ossington. The special remedial action of the mineral waters of Buxton would be out of the reach of many rheumatic sufferers, were it not for this institution.

Convalescent Homes have been opened at Shotley Bridge, Newcastle, Sandgate, Leeds "Ida" Home, and Liverpool Children's Home. Nursing institutes at Taunton, Sunderland, Newcastle, and elsewhere. Very many hospitals commenced in 1888 should be concluded this year; amongst which we note Dundee Fever Hospital, Glossop Small-pox Hospital, Glasgow Victoria Infirmary, Hertford Infectious Hospital, Leicester Children's Hospital, Merthyr Tydfil Hospital, Redruth Women's Hospital, Tynemouth Infirmary, and Wakefield Isolation Hospital.

At the quarterly meeting of the Leicester Infirmary governors on January 8th, the difficult subject of the admission and treatment of fever patients came up for discussion. It appears that last year there were 184 fever patients, who cost the infirmary £1,800. This is a grave financial difficulty which the governors cannot meet alone. It is therefore proposed to no longer admit fever patients free, but to make a small charge for all such cases. This will probably result in a large number of cases going to the fever hospital proper.

Great plans have been laid at Birmingham for the efficient working of the new infirmary, and the Guardians have been earnestly considering the past with a view to securing a good policy for their guidance in the future. Mr. Hardie, who for twelve years has shown himself to be a good administrator of Greenock Poor House and Asylum, has been elected the first master; and the Guardians have been fortunate in securing the services of Miss A. E. Gibson, who was for some years head of the large infirmary at Brownlow Hill, Liverpool, and who comes with proofs of very high qualifications for the position of matron. The medical staff will consist of a visiting physician, a visiting surgeon, two resident assistants, a clinical clerk (who must be also a qualified medical practitioner), and a dispenser. Although the present workhouse infirmary is so overcrowded, the medical work therein is of a very high order; but, with the enormous advantages of the new infirmary the Guardians look forward to still greater efficiency. The staff of nurses will be large, and, in addition, there will be about fifty other officers, attendants, and servants.

THE following vacancies are announced this week, the amount of salary in each case being given after the name of the institution :—

**Resident Medical Officers.**

Parish of Lyrie, Aberdeenshire. Medical Officer.
Rainhill County Asylum. Junior Assistant Medical Officer.—£105, board, etc.
Hospital for Sick Children, Great Ormond Street. Medical Registrar and Pathologist.—£50.
Birmingham General Hospital. Assistant House Surgeon.—Board, etc.
Jaffray Hospital, near Birmingham. Resident Medical Officer.—£150, board, etc.
Liverpool Eye and Ear Infirmary. House Surgeon.—£80, board, etc.
Mason Science College, Birmingham. Demonstrator in Physiology.
Chelsea Hospital for Women. Medical Officer.—£80, board, etc.
Dental Hospital of London. Dental Surgeon.
Hants County Asylum. Junior Assistant Medical Officer.—£100, board, etc.
Evelina Hospital for Sick Children. House Surgeon.—£70, board, etc.

**Non-resident Medical Officers.**

St. George's Hospital. Lecturer on Chemistry.
Victoria Hospital for Sick Children. Physician to In patients.
Sussex County Asylum. Physician and Assistant Physician.
Manchester Royal Infirmary. Honorary Assistant Physician.
Great Northern Central Hospital. Physician to Out-patients.

---

THE Cottage Hospital at St. Paul's Cray is able to record a year's useful work, and a satisfactory balance-sheet. Of the seventy-seven cases treated during the year, six were strangers or vagrants; so the hospital does not narrow its benefits to those who live near it, though these, of course, make the most frequent demands on it. The payments from patients amounted to £97 8s. 3d., and the total receipts were £555 19s. 9d., against an expenditure of £500 18s. 10d. The expenditure is £44 less than that of last year, although a larger number of patients have been treated—a fact which testifies to the care and ability of the matron, Miss Margaret Hordern. Miss Hordern has been successful in starting a Samaritan Fund, the value of which can scarcely be overrated.

---

AMONG the institutions outside London whose claim to its generosity is acknowledged by the fact of receiving a donation from the Hospital Sunday Fund is "The Firs" Home for Incurable Consumptives, at Bournemouth. Most hospitals for consumption take only cases where residence in a mild climate, and proper treatment, are likely to effect a cure. When patients are nearly, or quite, past hope of recovery, very few places are open to them. The Firs is one of these. The patients make a small payment, which last year amounted to £431 3s., but as the total cost of the Home was £1,261 12s. 1d., it is evident that it must rely to a great extent on the gifts of the charitable, and as it is an institution which does not confine its benefits to its own district, it should not be totally dependent on local support.

---

THE little books and pamphlets of the National Health Society are not so well known as they ought to be. If they were diffused with one quarter of the vigour with which religious tracts are spread, we should have a little less ignorance of sanitary laws amongst the inhabitants of our cottages and small tenements. The leaflets are more like small placards, and by their large print enforce attention. They deal with such subjects as measles, fresh air, &c., and cost ninepence per dozen. The penny books are small and handy, and often written in interesting dialogue. "A Pair of Small Villas" is the title of one, which in a narrative form contrasts the desirable with the undesirable dwelling. More ambitious books published by the society are Ernest Hart on "London, Old and New," Dr. Pope on "The Human Lamp," etc. To make popular and plain the simplest laws of health is certainly a worthy object, and many district visitors and others might avail themselves of some of these publications.

AT the late quarterly court of the governors of Addenbrooke's Hospital, the following donations were acknowledged: Mr. H. J. Banyard, of Grantchester, £100, which has been invested in Government Funds; £43 7s. 3d. from the Treasurer of the "Saul" Festival Fund; and £15 from Messrs. Keith and Tudor. The funds of the hospital are in a good way, and it is hoped to shortly build a new operating room and probationers' ward. The dental department of this hospital is now definitely established, and Mr. Rhodes and Mr. Jones have been elected honorary dental surgeons.

---

MR. CARR-GOMM writes to the *Times* to thank those who have responded to the quinquennial appeal on behalf of the London Hospital. The amount of support promised for the five years is £48,942, while that promised in 1883 was £61,000. The decrease is attributed to the fact that the People's Palace and other charitable undertakings have received most of the surplus cash of our well-known philanthropists. Mr. Carr-Gomm gives the following statistics of work done at the London last year: 8,268 in-patients were admitted, being eight in excess of the total of 1887. The daily number was 640, as against 644 in 1887, and the statistics now tell us that the mean residence of each patient was 27·59 days in 1888, as against 28·05 in 1887, or about half a day less—wonderfully close working—and a proof that the capabilities of the wards are very definitely tested. The mortality over all the cases was low, viz., 9·82. The total number of out-patients was 101,548, or 5,788 more than in 1887.

---

THE annual Christmas entertainment was given to the children of the Ladies' Charity School (instituted 1702), Powis Gardens, Notting Hill, by the Committee, on Friday last. About one hundred guests were present to witness the children going through their musical drill, for which they have had lessons during the past year from Mr. Sully, of the National Physical Recreation Society. After this performance a move was made to the school-room, where the girls recited and sang some pretty pieces, and at half-past four the business of the day began. Lieut.-Colonel R. A. Price, in a short speech, spoke of the advance made by the managers of the school during the year. It had been suggested to keep the girls twelve months longer in the school, and give them the advantage of more technical instruction. This had been carried out by giving lessons in dressmaking, cooking, and getting up fine linen, besides those in physical exercise (suggested last Christmas by Mr. Burdett, and given by a subscriber), the efficiency of which they had just had an opportunity of judging. He was sorry to say the institution found itself £30 in debt. It would be a pity to entrench on the funded property of the school, and he trusted the deficiency might be met by all present who were not subscribers becoming so. In thanking the Countess Amherst for so kindly coming to give the girls their prizes, he said she was one of the oldest supporters of the charity, having been a subscriber for nearly fifty years. The prizes were for house work, needlework, tidiness, and general industry, beside the usual ones for school-room work. Her Ladyship gave them with a few appropriate words to each girl, and concluded with a short speech, recommending them to make the most of the few years they had for learning, and hoped that they would by their conduct when in service show the good training they had received at school. The children then went downstairs, where a sumptuous tea was prepared for them, after which Aladdin with his wonderful lamp, to the great delight of the young people present, produced beautiful gifts for every one out of a glittering cave illuminated with coloured lights, the good genii being little Master Francis Burdett, the third son of the gentleman who had provided the money to purchase the presents for the school children.

